# The influence of hypoxia on tissue regeneration in oral and maxillofacial surgery – a systematic review

**DOI:** 10.1007/s00784-026-06808-9

**Published:** 2026-03-16

**Authors:** Albrecht H. F. Gäde, Eik Schiegnitz, Alexander W. Eckert, Keyvan Sagheb, Bilal Al-Nawas, Johannes R. Kupka

**Affiliations:** 1 Department of Oral and Maxillofacial Surgery, University Medical Center Mainz, Mainz, 55131 Deutschland; 2 Department of Oral and Maxillofacial Surgery, University Hospital of the Paracelsus Medical Private University, Nürnberg, Germany

**Keywords:** Bone regeneration, Tissue regeneration, Wound healing, Hypoxia, HIF

## Abstract

**Purpose:**

Hypoxia is an inevitable consequence of surgical interventions such as bone augmentation and soft tissue transplantation in oral and maxillofacial surgery. Cellular adaptation to hypoxic conditions critically influences regenerative processes, including osseointegration, angiogenesis and tissue integration. This systematic review investigated the effects of hypoxic conditions and hypoxia-regulating strategies on tissue regeneration, with the aim of identifying mechanisms to enhance clinical outcomes.

**Methods:**

Following the PRISMA guidelines, a systematic search was performed in MEDLINE (via PubMed), Cochrane, and Web of Science up to 31st May 2025, including reference list and citation screening. The risk of bias was assessed according to the SYRCLE risk of bias tool.

**Results:**

Of 5790 studies, 9 met the inclusion criteria. These studies investigated various interventions, including gene therapy targeting hypoxia-inducible factor 1α (HIF-1α), oxygen-releasing biomaterial scaffolds, hyperbaric oxygen treatment and hypoxia preconditioning of bone marrow mesenchymal stem cells. Some studies showed enhanced bone formation and vascularization with HIF-1α upregulation or hypoxic preconditioning, while others highlighted HIF-1α’s role in osteoclast activation and bone resorption. Hyperbaric oxygen treatment consistently improved bone healing.

**Conclusion:**

Current evidence highlights a complex interplay between hypoxia and regenerative outcomes in oral and maxillofacial surgery. Although modulation of HIF-1α and the hypoxic microenvironment hold promise, further research is needed to clarify optimal strategies for maximizing benefits and minimizing detrimental effects.

**Clinical relevance:**

The ability to influence the HIF pathway in beneficial manner may be a cornerstone to unlocking the next generation of regenerative therapies in oral and maxillofacial surgery.

## Background

**Fig. 1:**
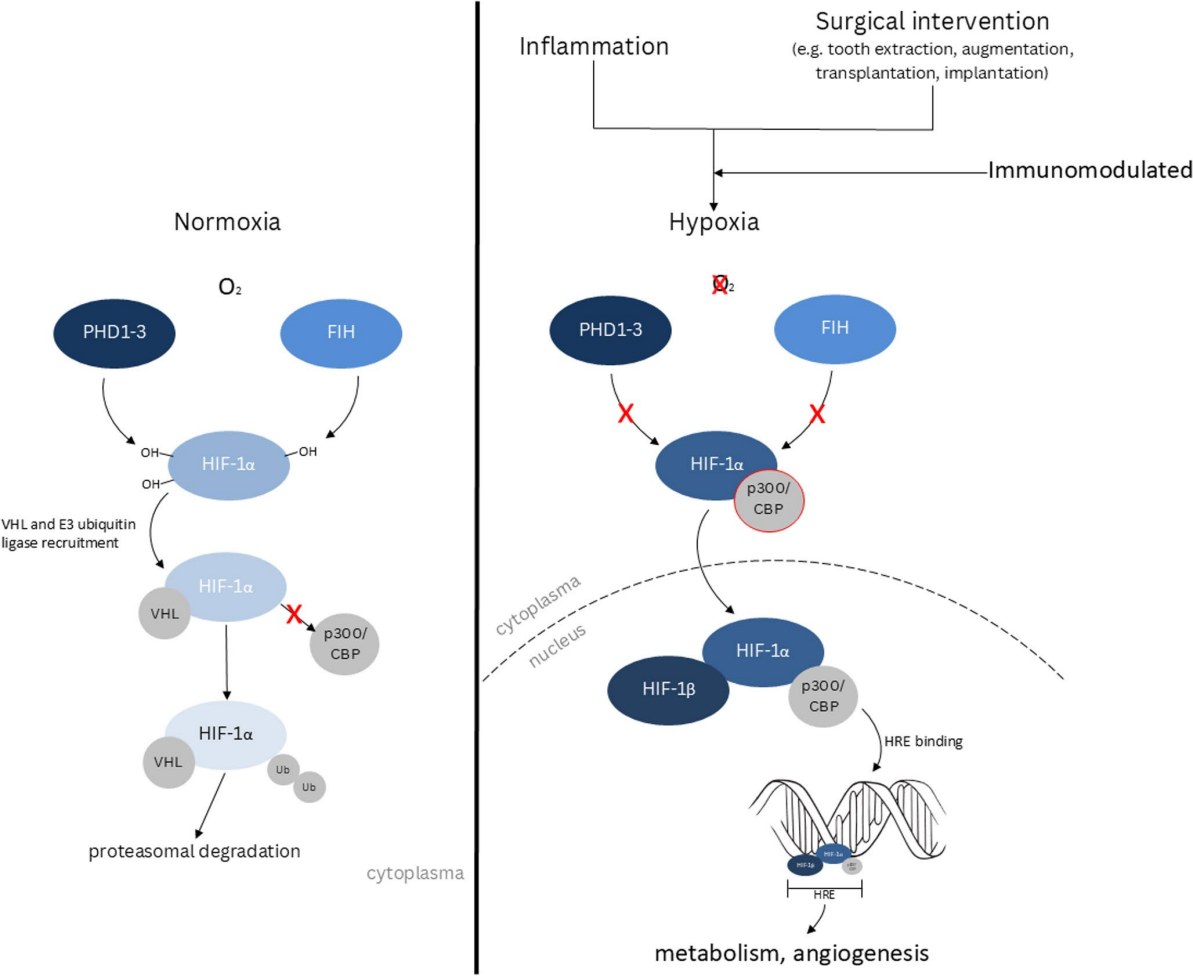
Schematic representation of HIF-1α regulation under normoxia (left) and hypoxia (right). On the left (normoxia), HIF-1α is hydroxylated by prolyl hydroxylases (PHDs) and FIH, which marks it for degradation via the VHL-dependent ubiquitin-proteasome pathway, preventing the transcription of hypoxia-inducible genes. On the right (hypoxia), PHDs and FIH are inactive, allowing HIF-1α to stabilize, translocate into the nucleus and form a transcriptional complex with HIF-1β and co-activators (p300/CBP). This leads to the expression of genes involved in metabolism and angiogenesis

Tissue regeneration in the context of oral and maxillofacial surgery remains a challenge, particularly in procedures involving augmentation, implant placement and tissue transplantation. After surgical interventions, the affected tissue undergoes a transient state of insufficient oxygen supply due to disrupted vascular supply and increased metabolic activity, which is defined as hypoxia. In transient state hypoxia is a key element of every healing process, as it induces the activation of the immune system, neovascularization and cell metabolism [1–3]. In contrast chronic hypoxia leads to ischemia. A key regulator of this adaptive response to low oxygen partial pressure are several isoforms of hypoxia-induced factor (HIF), a transcription factor orchestrating cellular adaption to hypoxia. The molecular mechanisms of hypoxic adaptation are described briefly below. A schematic overview of HIF-1α regulation by PHDs and FIH is provided in Fig. 1.

At the single-cell level, eukaryotic cells have developed the capability to sense changes in intracellular oxygen levels rapidly through a family of hydroxylases, specifically named prolyl-4-hydroxylase domain (PHD) proteins 1–3 [4–7]. These hydroxylases were first specified as oxygen sensors within the HIF pathway and belong to Fe(II)- and 2-oxyglutarate-dependent dioxygenase superfamily [8, 9]. The PHDs enable cells to adapt to hypoxia by modulating the of expression of specific genes involved in oxygen homeostasis [8, 10]. The dimeric HIF transcription factor is composed of α- and β-subunits. There are three known α (HIF-1α, HIF-2α, HIF-3α) and one HIF-β-subunit. Among these, the PHD-dependent regulation is described best for HIF-1α which is why the following description focusses on this subunit. Under normoxic conditions, PHD 1–3 hydroxylates two prolyl residues of HIF-1α, enabling recognition by the von Hippel-Lindau protein, which recruits an E3 ubiquitin ligase [8, 10, 11]. As a consequence, HIF-1α is polyubiquinated and degraded by proteasomes. The HIF-mediated enhancement of gene expression is thus limited. PHD require molecular oxygen as a co-substrate, consequently the availability of oxygen is crucial for their enzymatic activity [8]. In addition to the PHDs the factor-inhibiting hypoxia-inducible factor (FIH) acts as another oxygen-dependent regulator of HIF-1α. Like PHD 1–3 it belongs to the dioxygenase superfamily, but hydroxylates an asparagine residue [12–15]. This asparagine hydroxylation prevents the interaction of the histone acetyl transferases CBP/p300 with HIF-1α and as a result decreasing the impact of HIF-1α on specific genes [16–21]. Under hypoxic conditions the enzymatic activity of PHDs and FIH are diminished, resulting in a stabilization and accumulation of HIF-1α. As a result, it can migrate into the nucleus and form an active heterodimer with HIF-1β. This active form binds to the hypoxia response elements (HREs), initiating the transcription of a wide array of genes involved in diverse processes, including angiogenesis and energy metabolism [1, 10].

A deep understanding of the molecular regulators like HIF is increasingly critical for personalized treatment planning, improving augmentation and implant success rates and adapting regenerative innovations. Thus, this systematic review aims to summarize the current state of hypoxia research in oral and maxillofacial surgery with special focus on tissue regeneration, augmentation and implant placement, linking it with findings from other research fields and identify future research topics. It serves as a basis for future basic research in tissue regeneration as well as clinical research in oral and maxillofacial surgery and to identify relevant gaps for future translational research.

## Methods

This systematic review was guided by the PRISMA checklist and statement [22] and was registered in the International Prospective Register of Ongoing Systematic Reviews (PROSPERO) (ID: 628678).

The aim is to shed light on the following questions:

- What are the underlying mechanisms regarding bone augmentation, tissue regeneration and neovascularization?
- Are there points of contact for an individualized approach for tissue regeneration in oral surgery?
- What are possible targets for drugs minimizing the risk of ischemia in tissue regeneration?

For this purpose applicable PICO criteria were defined:

- P – Animals on which augmentative, orthognathic or implant oral or maxillofacial surgery was performed
- I – Exposure to hypoxia-related interventions (e.g. oxygen releasing scaffolds, HIF-1α stabilization, hypoxic preconditioning, hyperbaric oxygen therapy)
- C – Normoxic condition or no treatment regarding the HIF pathway
- O – Histological, biomechanical and radiographic measurements

The systematic electronic literature search was performed in the databases Medline (via PubMed), Cochrane and Web of Science. Detailed search terms are provided in Table 1. In addition, reference lists and citations were also screened for relevant studies. The last check was performed on 31st May 2025 and was documented by using a commercially available spreadsheet software (MS Excel). All results were collected by using the citation software Endnote 20. Duplicates and triplicates were excluded at this point.

**Table 1:** Search terms

Hypoxia		Oral- and maxillofacial surgery
Hypoxia *OR* Hypoxia-inducible factor 1 *OR* Hypoxia-inducible factor-1 *OR* vascular endothelial growth factor *OR* Vascular endothelial growth factor A *OR* VEGF *OR* VEGF-A *OR* neovascularization, physiological *OR* angiogenesis, physiological *OR* microcirculation *OR* microvascular blood flow *OR* microvascular circulation	*AND*	remodeling, bone *OR* bone turnover *OR* bone turnovers *OR* turnover, bone *OR* turnovers, bone *OR* alveolar ridge augmentation *OR* augmentation, alveolar ridge *OR* ridge augmentation, mandibular *OR* grafting, bone *OR* bone grafting *OR* transplantation, bone *OR* alveolar bone grafting

A.H.F.G. performed the initial literature search independently, which was subsequently checked by J.R.K. Study selection was conducted independently by both reviewers based on titles and abstracts. In cases of disagreement, discrepancies were resolved through discussion involving all authors until consensus was reached. All authors reviewed and agreed on the final selection of included studies. Data extraction was performed by A.H.F.G. and subsequently reviewed by J.R.K.

The overall goal of this review was to provide a comprehensive overview of this topic. Therefore inclusion and exclusion for all studies were defined in accordance with Table 2.

**Table 2:** Inclusion and exclusion criteria

Inclusion	
I1: type of publication	Non-clinical studies, laboratory research, animal studies, in vitro and in vito studies, post-mortem studies
I2: Type of surgery	augmentative surgery, implant surgery, orthognathic surgery
I3: Language	Englisch, German
Exclusion	
E1: type of publication	Observational studies, randomized controlled trials, controlled and uncontrolled trials, systematic reviews with or without meta-analysis
E2: Language	Any other language aside from English and German

Only studies that investigated the influence of hypoxia on success in oral and maxillofacial surgery, especially augmentative, implant surgery and orthognathic surgery were included. The broad search terms explain the large number of studies that could be excluded, because they are thematically not relevant for the aim of this review. All included studies were assessed regarding their risk of bias according to the SYRCLE risk of bias tool (Table 3) [23].

**Table 3:**
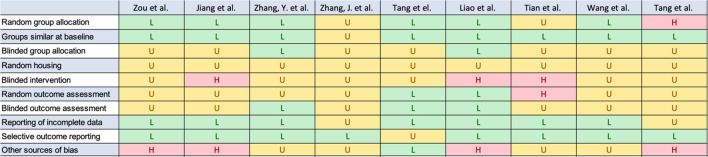
Risk of bias assessment according to the SYRCLE risk of bias tool

*H* High-risk, *L* Low-risk, *U* Unclear

**Fig. 2:**
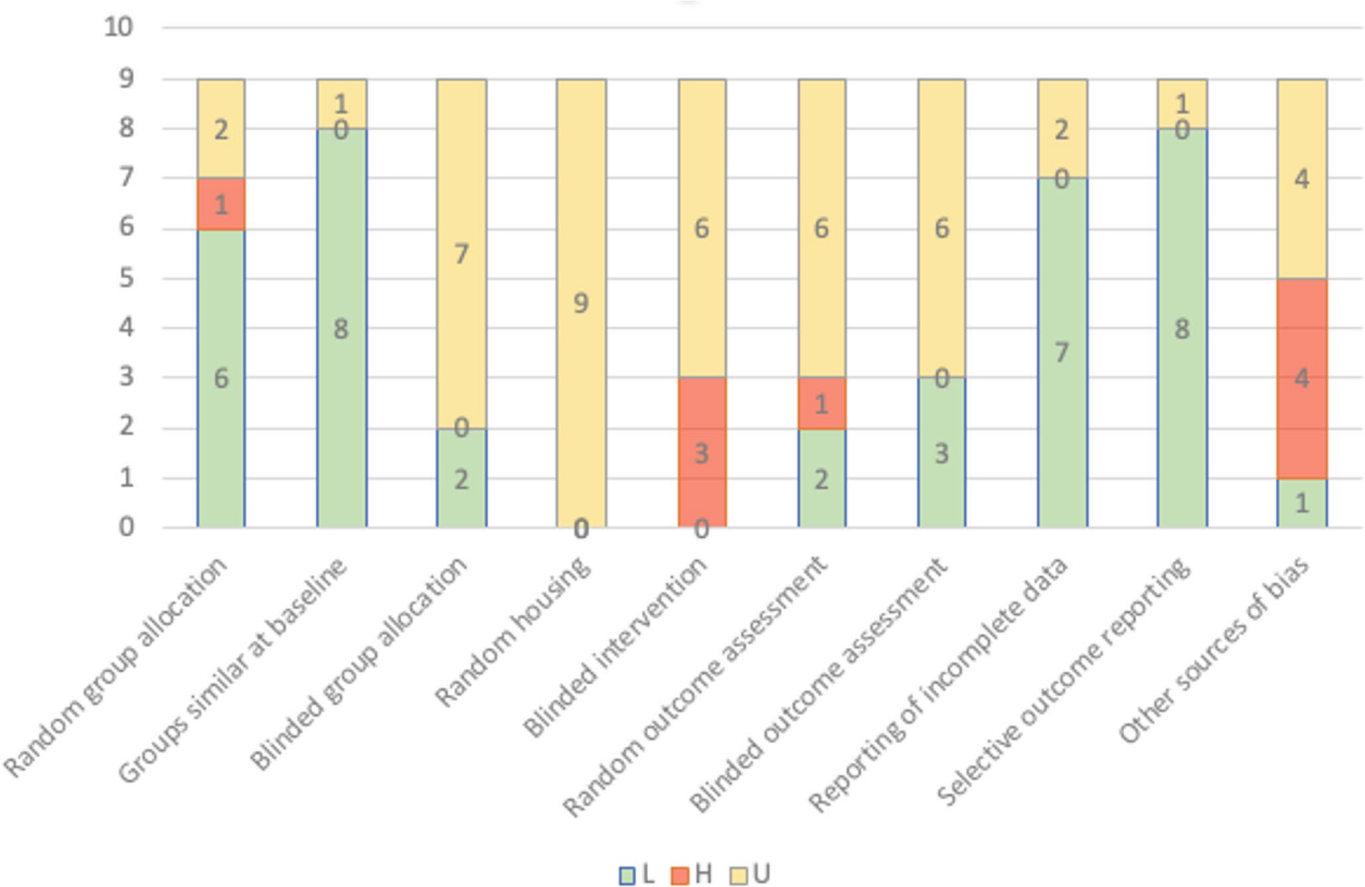
Overall bias levels across studies. H: High-risk; L: Low-risk; U: Unclear

## Results

**Fig. 3:**
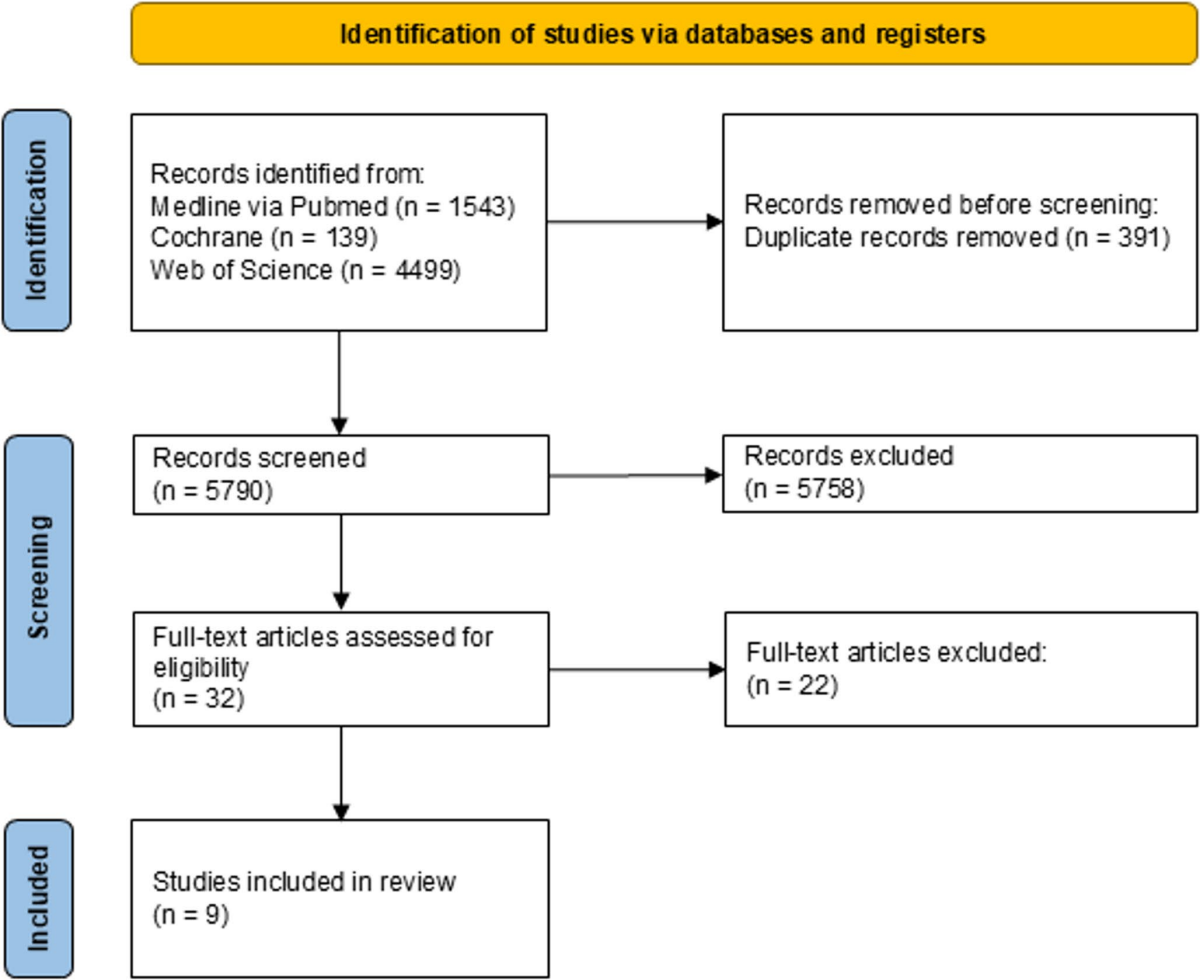
PRISMA flow chart

The first search yielded in 6181 results. After excluding duplicates, it reduced to 5790. Because of the very general search terms, a lot of publications were thematically far away from the desired topic (Fig. 3). After the title and abstract screening 32 publications were suitable for a full text screening.

Even though fifteen studies examined the connection between bone and hypoxia, they were excluded because they don’t have a direct link to oral and maxillofacial surgery [24–38]. Three studies were excluded because they investigated the correlation between hypoxia and the periodontal diseases [39–41]. One study was excluded because of the investigation of general hyperbaric oxygen treatment on bone metabolism [42]. Two studies were excluded due to the secondary role played by hypoxia in their study design [43, 44]. One study was excluded because it is a clinical study [45]. Nine studies remained that fulfilled the inclusion criteria [46–55]. Although the overall risk of bias was assessed low, several domains were rated as unclear or high risk, particularly regarding random housing, blinded intervention and blinded outcome assessment (Table 3). In addition to Table 3, an overview figure was generated to visualize the distribution of low, unclear and high risk of bias across SYRCLE domains for all included studies (Fig. 2).

Overall, the nine included studies demonstrated a dual and context-dependent role of hypoxia and HIF-1α in oral and maxillofacial bone healing. Five studies primarily reported beneficial effects of HIF-1α activation on tissue regeneration, particularly in scaffold-based approaches, distraction osteogenesis, hypoxic preconditioning of mesenchymal stem cells and hyperbaric oxygen therapy, showing enhanced bone formation, vascularization and implant osseointegration. In contrast, four studies highlighted HIF-1α–mediated osteoclast activation and bone resorption showing its role in bone remodeling. Together, these findings indicate that HIF-1α signaling can either promote bone formation or enhance bone resorption depending on the biological context.

Table 4 summarizes the key characteristics of the reviewed studies. Subsequently, their main results are presented in detail.

**Table 4:** Preclinical studies

	Test subjects	Surgical intervention	Intervention regarding the HIF-pathway	Outcome	
Zou et al. (2012)	Labrador Retrievers	- Seeding transduced BMSCs onto CMPC scaffolds to create cell-**scaffold** constructs - Immediate dental implantation with cell/scaffold constructs in mandibular defects	- Lentiviral transduction of BMSCs to express HIF-1α (Lenti-cHIF, Lenti-HIF) or GFP (Lenti-GFP control) - Cells seeded on CMPC scaffolds	µCT analysis: - BV/TV, Tb.Th, Tb.N, Bone mineral density (BMD) - histological analysis	**Scaffold**-based modulation of the HIF-pathway
	Nude Mice	- Subcutaneous implantation of cell/**scaffold** constructs		Ectopic bone formation	
Zhang et al. (2016)	30 male 6-week-old Sprague–Dawley rats (weighing 200–250 g)	gelatin sponge **scaffolds** implanted in alveolar bone defects	gene therapy with adenovirus encoding hypoxia-inducible factor-1α (AdHIF-1α) delivered via the gelatine sponge - Group 1 (Control): untreated defect - Group 2: scaffold + AdRFP - Group 3: scaffold + AdHIF-1α	µCT analysis: - BV/TV, Tb.Th, Tb.N Histological and immunhistological analysis: - expression levels of VEGF, bFDF	
Wang et al. (2022)	New Zealand white rabbits (male, 2.5 ± 0.5 kg)	3D-printed bionic oxygenated **scaffold** (PCL/nHA/CaO₂) implanted into 7 mm diameter cranial defects	- Group 1: 3D-printed bionic oxygenated caffolds were implanted into 7 mm diameter cranial defects - Group 2 (control): defects left empty or treated with non-oxygenated scaffolds	µCT analysis: - BV/TV, Tb.N	
Tang et al. (2023)	Mice	Bilateral Mandibular Osteotomy	HIF-1α knockout (CKO) and wild-type mice	µCT analysis: - bone volume (BV/TV) - osteoclast activity (TRAP staining) - expression of MMP-9, VEGF and CD31 were assessed.	**Osteotomy**-associated, HIF-mediated bone remodeling and repair
Tian et al. (2022)	mice	Mandibular Osteotomy	HIF-1α-conditional-knockout (Cko) and wild-type (Ctrl)	- µCT analysis: (BV/TV, Tb.Th, Tb.Sp, density, Tb.*N*) - expression at mRNA and protein level: HIF-1α and CT-1	
Zhang et al. (2018)	Sprague-Dawley Rats	Implantation of gelatin sponge (GS) with or without hypoxia preconditioned BMSCs into mandible defects (5 mm)	Hypoxia preconditioned BMSCs (with 0.5 mM DMOG under 1% O2 Condition)	µCT analysis: bone formation histological evaluation immunohistochemistry	
Jiang et al. (2015)	New Zealand White Rabbits	Mandibular distraction osteogenesis	Local rHIF-1α Administration - Group A: Saline Injection - Group B: 10 µg rHIF-1α Injection - Group C: 20 µg rHIF-1α Injection - Injections daily for 10 days	Protein Expression: Western Blot (BMP-2, bFGF, IGF, VEGF, RUNX2) Bone Formation: RBI uptake, CT analysis, DXA (BMD/BMC), Radiography Histology: H&E staining Mechanical Strength: 3-point bending.	
Tang et el. (2018)	Six-month-old female New Zealand white rabbits (2.3 ± 0.2 kg) RAW264.7 murine macrophages	In vivo: Mandibular osteotomy In vitro: RANKL-induced osteoclast differentiation in RAW264.7 cells, with and without hypoxia (2% O2 or CoCl2) and/or siHIF-1α transfection or inhibitors of glycolytic enzymes (CoA, levamisole, hypoxanthine and NaF)	In vivo: The control group received sham surgery without osteotomy. In vitro: siHIF-1α transfection	In vivo: - Histological analysis (H&E, TRAP, Toluidine Blue staining) to assess bone resorption, osteoclast number and morphology. - Immunohistochemical analysis (HIF-1α, LDHA, GCK, PKM2, PFK1, CTSK) to evaluate protein expression In vitro: - Osteoclast differentiation and activity: TRAP staining, toluidine blue resorption assay, phalloidin staining for cell size. - Gene expression analysis (RT-qPCR) of osteoclast markers (TRAP, CTSK, MMP-9) and glycolytic enzymes. - Protein expression analysis of HIF-1α and glycolytic enzymes. - Acid production and secretion: AO-EB staining, lactic acid and pyruvate measurements	
Liao et al. (2019)	32 beagle dogs	- **Extraction** of **lateral incisors of** the **maxillary and mandible** of each dog - Right upper and lower incisor extraction sockets (A2C2) allowed to heal naturally - Left upper and lower incisor sockets (B2D2) received implants of a commercial bone substitute	- HBO group: hyperbaric oxygen (100% O2​, 2.4 atm, 90 min/day, 5 times/week, 6 weeks) - NBO group: normobaric oxygen (normal air in the same chamber)	- Clinical observation - Cone-beam computerized tomography (CBCT) - Histomorphology observation - Expression levels of VEGF - Expression levels of BMP-2	HBO treatment

### Scaffold-based modulation of the HIF-pathway

Zou et al. examined the effects of HIF-1α-transduced bone marrow mesenchymal stem cells (BMSCs) on bone regeneration and osseointegration on immediate dental implantation with cell/scaffold constructs in mandibular defects [55]. Using a lentiviral vector, a constitutively active HIF-1α was overexpressed in BMSCs and implanted into peri-implant bone defects. micro-computed tomography (µCT), histological analysis and immunohistochemistry revealed significantly increased bone volume fraction (BV/TV), bone mineral density (BMD) and bone-to-implant contact (BIC) in experimental groups (*p* < 0.01). Fluorescent labeling confirmed enhanced mineralization and immunohistochemistry demonstrated sustained HIF-1α expression. The study suggests that HIF-1α-modified BMSCs could improve osseointegration and implant stability in cases of insufficient bone volume.

Zhang et al. investigated the effects of HIF-1α gene therapy on angiogenesis and osteogenesis in alveolar bone defect regeneration using a rat model [53]. The study employed a gelatin scaffold impregnated with an adenovirus encoding HIF-1α (AdHIF-1α) and compared it to untreated defects and scaffolds containing a control adenovirus (Adenovirus encoding red fluorescent protein, AdRFP). Results from µCT and histological analysis demonstrated a significant increase in bone volume (BV/TV), trabecular thickness (Tb.Th) and vessel formation in the AdHIF-1α group compared to controls. Immunohistochemical analysis revealed significantly elevated expression of vascular endothelial growth factor (VEGF) and basic fibroblast growth factor (bFGF), supporting the role of HIF-1α in promoting vascularization. The statistical analysis confirmed significant differences (*p* < 0.05) in bone and vessel formation between the treatment and control groups. The findings suggest that gene therapy using AdHIF-1α enhances alveolar bone regeneration by promoting coupled angiogenesis and osteogenesis.

Wang et al. developed a 3D-printed bionic oxygenated scaffold to enhance bone regeneration under hypoxia [47]. The scaffold, integrating polycaprolactone (PCL), nanohydroxyapatite (nHA) and gelatin-calcium peroxide (CaO₂) microspheres, sustained oxygen release for over two weeks, improving BMSC survival, proliferation and osteogenic differentiation by downregulating HIF-1α and upregulating RUNX2, BMP2 and OCN. In a rabbit calvarial defect model, µCT and histological analyses showed significantly increased bone volume and trabecular number, with compressive strength reaching 1.5 GPa after 12 weeks. Immunohistochemistry confirmed enhanced proliferation (Ki67, PCNA) and reduced apoptosis.

### Osteotomy-associated, HIF-mediated bone remodeling and repair

Jiang et al. investigated the effects of HIF-1α on bone regeneration during distraction osteogenesis in a rabbit model [54]. Fifty-one rabbits underwent mandibular lengthening and received either 20 mg (Group C) or 10 mg (Group B) of recombinant HIF-1α or saline (Group A). Radionuclide bone imaging showed significantly higher uptake in Groups B (*P* = 0.013) and C (*P* < 0.001) compared to Group A. Bone mineral density and content were highest in Group C at 4 and 8 weeks. Mechanical testing demonstrated superior bone strength in Group C (289.71 ± 43.31 N) versus Group A (194.94 ± 26.24 N, *P* < 0.001) and Group B (240.52 ± 36.22 N, *P* = 0.012). Radiographic and histological analyses confirmed enhanced bone formation in HIF-1α-treated groups. The findings suggest HIF-1α as a promising agent to accelerate osteogenesis and mineralization in distraction osteogenesis.

Tang et al. (2019) investigated the effects of mandibular osteotomy-induced hypoxia on osteoclast activation and bone resorption, focusing on the role of HIF-1α and glycolysis [51]. Using a rabbit mandibular osteotomy model and hypoxia-exposed RAW264.7 murine macrophages, they found that osteotomy led to increased HIF-1α expression and upregulation of glycolytic enzymes (LDHA, GCK, PKM2, PFK1) in osteoclasts. Hypoxia promoted osteoclast differentiation, acid secretion and bone resorption, which were significantly reduced by HIF-1α knockdown and glycolysis inhibition. Statistical analyses confirmed these findings, with significant increases in osteoclast surface area, TRAP-positive cells and lactic acid production under hypoxic conditions (*p* < 0.05). The study concludes that HIF-1α-driven glycolysis is a key adaptive mechanism facilitating alveolar bone remodeling post-osteotomy.

Tang et al. (2023) investigated the role of HIF-1α in the regional acceleratory phenomenon (RAP) following bilateral mandibular osteotomy in mice [46]. Using HIF-1α conditional knockout (CKO) models and in vitro hypoxia-mimicking experiments with RAW264.7 cells, the study demonstrated that osteotomy induced HIF-1α expression. This, in turn, promoted osteoclastogenesis and alveolar bone resorption via dendritic cell-specific transmembrane protein (DC-STAMP)-mediated cell fusion. µCT and histological analyses revealed significantly reduced bone resorption and osteoclast numbers in CKO mice, confirming HIF-1α’s critical role in RAP. Statistical analysis showed significant differences in BV/TV and connectivity density (Conn.D) between control and CKO groups (*p* < 0.05), with peak osteoclast activity occurring at day 10 post-osteotomy. In vitro, CoCl₂-induced hypoxia increased DC-STAMP expression and osteoclast fusion, effects that were suppressed upon HIF-1α silencing. These findings highlight HIF-1α as a key regulator of osteotomy-induced RAP, linking hypoxia to osteoclast-mediated bone remodeling.

Tian et al. examined the role of HIF-1α in osteoclast activation and mandibular bone repair [48]. Using osteoclast-specific HIF-1α knockout (CKO) mice, they found that HIF-1α enhances osteoclast differentiation and resorption, promoting bone healing via cardiotrophin-1 (CT-1). CKO mice exhibited reduced osteoclast activity, lower CT-1 expression and delayed bone regeneration, as evidenced by decreased BV/TV and Tb.Th and increased Tb.Sp​ Statistical analysis confirmed significant differences (*p* < 0.05)​.

Zhang et al. investigated the repair of critical-sized mandibular defects in aged rats using hypoxia-preconditioned BMSCs with upregulated HIF-1α [52]. Hypoxia preconditioning (1% O₂, 0.5 mM dimethyloxaloylglycine (DMOG) enhanced BMSC survival under ischemic conditions (*p* < 0.01) and significantly increased angiogenesis (VEGF expression, tube formation, *p* < 0.01) and osteogenesis (ALP activity, mineralization, *p* < 0.01). In vivo, µCT analysis showed greater bone regeneration in the preconditioned BMSC group (BV/TV: 36.87%) compared to controls (*p* < 0.05). Immunohistochemistry confirmed increased vascularization (CD31, VEGF) and osteogenic markers (Runx2, OCN). These findings suggest that hypoxia preconditioning improves BMSC-mediated bone healing in aged individuals.

### Alveolar ridge preservation and post extraction wound healing under hyperbaric oxygen therapy

Liao et al. examined the effects of hyperbaric oxygen (HBO) on bone healing and alveolar ridge preservation after tooth extraction in beagle dogs [50]. Compared to normobaric oxygen (NBO), HBO treatment (100% O₂, 2.4 atm, 90 min/day, 5 days/week, 6 weeks) significantly reduced bone resorption and enhanced new bone formation (*P* < 0.05). Cone-beam CT and histological analysis showed greater bone mineral density and alveolar ridge preservation in the HBO group. Immunohistochemistry confirmed increased VEGF and BMP-2 expression, indicating improved angiogenesis and osteogenesis. Statistical analysis confirmed significant differences in bone width and height reduction (*P* < 0.001). The findings suggest HBO accelerates post-extraction healing and could improve clinical outcomes in dental implantology.

## Discussion

### The influence of a hypoxic microenvironment on tissue regeneration

Adequate oxygen supply plays a key role in every tissue for physiological homeostasis. Nevertheless, surgical intervention, inflammation or neoplasia leads sooner or later to a hypoxic microenvironment in the affected tissue [56–59]. In the context of wound healing, tissue oxygen partial pressure may decline substantially and has been reported to reach levels as low as 0–2% [60–62]. Therefore, mammal cells developed diverse mechanisms to adapt their metabolism to the current state of available oxygen. Moreover, links to the immune system are also evident, as mentioned by Halligan et al. and Jucht et al. [6, 63].

It was shown that hypoxia significantly affects alveolar bone remodeling, bone mineral density and socket healing [64, 65]. The duration of the hypoxic environment is crucial for understanding the influence of hypoxia on bone. Siques et al. showed that long-term and short-term hypoxia differently affect bone tissue [66]. If the hypoxic condition becomes chronic it results in the loss of the augmentation [67–69]. In contrast, a short-term hypoxia in vivo has been shown to stimulate bone formation trough upregulation of HIF-1α regulated genes affecting angiogenesis and the activity of osteoblasts [65]. At this point it must be stated that outcomes of short-term hypoxia on osteogenic differentiation of stem cells are heterogeneous. Some studies show enhanced osteogenic differentiation and alveolar bone defect repair [70], while other studies revealed bone resorption as consequence of hypoxic treatment [71, 72]. This is probably due to different methods. From a clinical perspective, these findings suggest that strategies leveraging controlled hypoxia could enhance bone augmentation outcomes. The incorporation of biomaterials designed to regulate oxygen tension or promote HIF-1α activation may optimize bone graft integration and improve the success rates of bone augmentation procedures. However, further studies are needed to refine the optimal degree and duration of hypoxia required to maximize the success rate bone regeneration without compromising mineralization.

### Overview of relevant studies

A detailed reflection on the studies is presented in the following paragraph. Zou et al. investigated the therapeutical potential of HIF-1α-overexpressing canine BMSCs in bone repair [55]. The study provides experimental evidence in a preclinical large animal model concerning the potential applications of HIF-1α in promoting new bone formation and osseointegration of immediate implantation for oral function restoration. As a big concern in gene-mediated stem cell therapy is safety, the authors also focused also on neoplasia induced by HIF-1α-overexpressing canine BMSCs. The authors found no evidence for any tumors related to the transplanted BMSCs over the period of 12 weeks. Similar results were shown by Rajagopalan et al. They used a constitutively active HIF-1α transgene to treat critical limb ischemia patients [73]. To firmly confirm safety of HIF-1α-transduced BMSCs it will need long-term observations in vivo. Another approach to overcome the limitations of DNA expression plasmids and the chance of integration into host cell nuclear DNA and express the encoded gene indefinitely is to transfer biological active mRNA. This transfer technique is used broadly in cancer and Covid19 vaccines [74]. The advantage of this technique is that transfected mRNA does not travel to the nucleus, allowing fast translation and predictable degradation of the transcript over time [75–77].

The following two studies focused on the effect of HIF-1α on bone regeneration after osteotomy. Jiang et al. hypothesized that HIF-1α could promote the distraction osteogenesis (DO) process by simultaneously enhancing osteogenesis and angiogenesis [54]. The recombinant HIF-1α protein was applied by local injection into the distraction gap every 10 days. For clinical application the ideal dosage and delivery method need further investigation. An in vitro study by Wlodarczyk et al. investigating the influence of recombinant HIF-1α mRNA on primary human dermal fibroblasts [78]. They were able to show a rapid upregulation of all five downstream response genes (VEGF, PGF, ANGPT1, FLT1, EDN1) tested in this study. Although the chief concern of this study was not the regeneration of the bone, it still shows the effectiveness of this kind of treatment to promote the survival of surgical pedical flaps. This has been the focus of researchers for many decades [79–81].

The findings of Zhang et al. corroborate previous studies that have demonstrated the ability of HIF-1α to markedly enhance both vessel number and bone regeneration in ischemic tissue following local injection of HIF-1α vectors [53]. While the application of HIF-1α in angiogenesis and osteogenesis within tissue-engineered bone using adenoviruses and a gelatin scaffold is novel, the study aligns with existing research demonstrating the potential of HIF-1α to promote osteogenesis and mineralization.

At a first glance, one would like to think that the study by Wang et al. negates the importance of HIF-1α as mediator for regeneration. They introduced a 3D printed bionic PCL/nHA/CaO_2_ scaffold for bone regeneration [47]. With this approach they stimulate BMSC proliferation by making O_2_ available in the area of the defect [82]. The challenge is to provide enough oxygen with a constant release over a longer period of time, ideally over weeks or months, to overcome the hypoxia problem. On the other hand, a rapid oxygen release would lead to local cellular oxygen toxicity, as Colares et al. have shown in a RCT [83]. 3D-printing is already an established tool to improve the workflow, cost effectiveness and the patient related outcome and will gain more and more popularity in the future [84–86].

In their animal study from 2023 Tian et al. aimed investigate the key function of HIF-1α in osteoclasts and protein and mRNA levels of CT-1 after mandibular osteotomy [48]. They used osteoclast-specific HIF-1α-conditional-knockout (CKO) mice and a wild type as control group. The disadvantage of this study is missing information regarding age, sex and group size. To detect possible isolated effects, this essential information should be provided. HIF-1α-knockout primary osteoclasts inhibited bone resorption, delayed bone repair in the CKO-mice and the inhibition of HIF-1α-knockout primary osteoclasts osteogenesis via the coupling factor CT-1. These findings are consistent with other studies investigating the influence of HIF-1α on osteoclast [87, 88].

Tang et el. focused on the functional link between metabolism and hypoxic response, briefly between hypoxic osteoclastogenesis, bone resorption activity and glycolysis after mandibular osteotomy [46]. The study only focuses on one cell type: osteoclasts. For a more comprehensive understanding of the interplay between all cell types involved in bone remodeling further studies need to be carried out in this regard. Other diseases with a disturbed homeostatic relationship resulting in pathological bone loss can benefit from these findings as well. There is evidence that overactivation of osteoclasts is directly responsible for osteoporosis [89], cancer metastases in the bone [90] and rheumatic arthritis [91, 92], just like that HIF-1α is required for osteoclast activation [93, 94].

Liao et al. used hyperbaric oxygen treatment (HBO) to investigate its role in alveolar ridge preservation in the healing of extraction sockets [50]. The split mouth study design delivers robust results with significant difference in new bone formation (*P* < 0.05) and bone mineral density (*P* < 0.05) between the HBO and NBO groups. Furthermore, the HBO group showed significantly greater new bone and bone reconstruction based on histology with increased levels of VEGF and BMP-2. Hollander et al. were able to show similar results regarding HBO for wound dehiscence after intraoral bone grafting in a case series [95]. As mentioned before osteoclasts are activated by a hypoxic environment. Consequently, HBO should reduce osteoclast formation, increase osteoblast proliferation and differentiation and thus promote new bone formation [96].

Zhang et al. investigated the regenerative potential of hypoxic preconditioned BMSCs on critical sized mandibular defects in aged rats [52]. Furthermore, they were able to show that the treatment of BMSCs with DMOG combined with hypoxic treatment shows an upregulation of HIF-1α in BMSCs und enhanced survival rates in vitro. These results could be of particular interest in regenerative medicine and especially in older patients as there are fewer osteoblasts in aged bone compared to young bone [97, 98].

Tang et al. investigated the role of HIF-1α in postoperative bone resorption following bilateral mandibular osteotomy [46]. They were able to show that HIF-1α regulates the expression of DC-STAMP, which plays an important role for the fusion of osteoclast precursor cells and consequently plays a central role in bone resorption [99]. To provide deeper understanding of the complex mechanism of bone hemostasis and the interaction with the immune system, future studies should focus on translating these findings to humans. These pilot studies should aim to monitor HIF-1α expression in human bone tissue in combination with relevant molecular markers such as VEGF, DC-STAMP, CTX and TRAP5. Particularly the postoperative dynamics of HIF-1α activation should be investigated, clarifying how long active HIF signaling persists and how it evolves during the different phases of healing. Additionally, an analysis of patient subgroups with systemic risk factors like diabetes or osteoporosis can be beneficial to determine an altered hypoxia response in these individuals. This approach aligns with the growing demand for personalized and individual therapeutic strategies by accounting for patient-specific variability in hypoxia responses and regenerative potential [100].

## Future perspectives

Building on the aim of this review, the available preclinical evidence demonstrates that controlled modulation of the hypoxic microenvironment and HIF-related pathways holds considerable potential to enhance tissue regeneration in oral and maxillofacial surgery. In particular, experimental approaches combining biomaterial-based strategies with local oxygen modulation have shown promising regenerative outcomes in animal models.

Among these, oxygen-releasing, 3D-printed scaffold systems integrating materials such as polycaprolactone and nanohydroxyapatite represent a promising direction for future research [101–105]. These approaches may allow a more precise spatial and temporal control of oxygen availability within bone defects and could contribute to improve regenerative outcomes while avoiding additional surgical procedures associated with non-resorbable materials. However, further translational studies are required to define optimal scaffold composition, oxygen releasing kinetics and long-term biological effect under clinically relevant conditions. Beyond bone regeneration, future research should also address the role of hypoxia in soft tissue healing, as adequate vascularization and mucosal stability are critical for long-term success in regenerative, augmentative and implant procedures. Hypoxia-regulated pathways influence angiogenesis, fibroblast activity and epithelial wound healing, highlighting the need for integrated hard- and soft-tissue-orientated regenerative strategies. In this context, non-invasive techniques such as endoscopic hyperspectral imaging may provide valuable tools for assessing tissue perfusion and oxygenation in clinical settings, although further validation in compromised patient populations is required [106, 107]. Overall, future studies should focus on standardized experimental models, improved methodological rigor and translationally relevant outcome measures to bridge the gap between preclinical findings and clinical application to hypoxia-based regenerative therapies in oral and maxillofacial surgery.

## Limitations

Certain limitations of the present review should be acknowledged.

First, the present systematic review is limited by the exclusively preclinical nature of the available evidence. Randomized clinical trials were not included, as, to the best of the authors knowledge, no valid randomized clinical studies have yet examined hypoxia- or HIF-1α-related interventions on tissue regeneration in oral and maxillofacial surgery. Consequently, this review was designed to synthesize experimental evidence to establish a mechanistic und biological foundation that may support future translational and clinical research. Furthermore, the literature search was restricted to peer-reviewed and indexed publications to ensure methodological quality and reproducibility. The exclusion of grey literature, conference abstracts and non-indexed studies may have introduced a degree of publication bias. Second, the heterogeneity among the included studies regarding experimental models, surgical approaches, hypoxia-related interventions and outcome parameters needs to be addressed. The reviewed strategies ranged from genetic modulation of HIF-1α and hypoxic preconditioning of cells to oxygen-releasing biomaterials and hyperbaric oxygen therapy, while assessed outcome included histological, molecular, biomechanical and radiographic measurements. This methodological diversity precluded quantitative synthesis and limits the ability to derive generalized conclusions concerning the optimal modulation of hypoxia for regenerative purposes. Third, many studies focused on isolated molecular or cellular mechanisms, often emphasizing either osteogenic or osteoclastic pathways. As a result, the complex interactions between different cell types in bone remodeling, angiogenesis, inflammation and immune regulation could be only partially addressed.

Although the overall risk of bias was assessed as low according to the SYRCLE risk of bias tool, several domains were rated as unclear or high risk across individual studies, particularly regarding random housing, blinded intervention and blinded outcome assessment (Table 3). These limitations may increase the risk of detection bias. Therefore, treatment effects may be overestimated in some studies.

## Conclusion

Hypoxia and the HIF pathway play a central role in regenerative processes in oral and maxillofacial surgery. This is especially relevant given the dual challenge of regeneration both bone and soft tissue at the same time. Current preclinical evidence shows that controlled modulation of hypoxia through oxygen-releasing scaffolds, gene therapy, or hyperbaric oxygen therapy can enhance bone regeneration and vascularization. However, the effects are highly context-dependent and overactivation of HIF-1α may lead to bone resorption. Future strategies should focus on precise control of oxygen levels in the effected tissue, improved diagnostics (e.g. eHSI) and oxygen releasing biomaterials to locally influence hypoxia signaling. The ability to influence the HIF pathway in beneficial manner for tissue regeneration may be a cornerstone to unlocking the next generation of regenerative therapies in oral and maxillofacial surgery.

## Data Availability

All data generated or analyzed during this study are included in this published article.

## Abbreviations

AdHIF-1α: Adenovirus encoding Hypoxia inducible factor 1
AdRFP: Adenovirus encoding red fluorescent protein
ALP: Alkalic phosphatasis
ANGPT1: Angiopoietin 1
bFGF: Basic fibroblast growth factor
BIC: Bone implant contact
BMD: bone mineral density
BMD/BMC: Bone mineral density/bone mineral content
BMP2: Bone morphogenetic protein 2
BMSCs: Bone marrow mesenchymal stem cells
BV/TV: Bone volume to total bone volume ratio
CaO₂: Calcium peroxide
CBCT: Cone beam computed tomography
CD-31: Cluster of Differentiation 31
CKO: Conditional knock out
CMPC: Calcium-magnesium phosphate cement
Conn.D: Connectivity densisty
CoCl_2_: Cobalt(II)-cloride
CT-1: Cardio tropin 1
CTSK: Cathepsin K
DC-STAMP: Dendritic cell specific transmembrane protein
DMOG: Dimethyloxalylglycine
DO: Distraction osteogenesis
FIH: Factor inhibiting hypoxia inducible factor
FLT1: Fms-like tyrosine kinase 1 (= Vascular Endothelial Growth Factor Receptor 1)
GCK: Glucokinasis
HBO: Hyperbaric oxygen
HE: Hematoxylin eosin staining
HIF: Hypoxia inducible factor
HRE: Hypoxia response element
IGF: Insulin-like growth factor
Ki67: Proliferation marker
LDHA: Lactatdehydrogenase-A
NBO: Normobaric oxygen
nHA: Nanohydroxyapatite
OCN: Osteocalcin
PCL: Polycaprolacton
PCNA: Proliferating Cell Nuclear Antigen
PFK1: Phosphofructokinase-1
PGF: Prostaglandin factor
PHD: Prolyl hydroxylases
PKM2: Pyruvatkinase 2
RAP: Regional accelerator phenomenon
RUNX2: Runt-related transcription factor 2
Tb.N: Trabecular number
Tb.Sp: Trabecular separation
Tb.Th: Trabecular thickness
VEGF: Vascular endothelial growth factor
µCT: Micro-computed tomography
